# Electric field distribution models in ECT research

**DOI:** 10.1038/s41380-022-01516-8

**Published:** 2022-03-18

**Authors:** Alexander Sartorius

**Affiliations:** Department of Psychiatry and Psychotherapy, Central Institute of Mental Health (CIMH), Medical Faculty Mannheim, University of Heidelberg, Mannheim, Germany

**Keywords:** Depression, Neuroscience

## To the Editor:

The work of Deng et al. [1, 2] is in many aspects a very remarkable and outstanding study. Without a doubt, it makes a very important contribution to, among other things, the issue of electric current and electrode position in ECT.

My intention in writing this letter to the editor is not so much as a psychiatrist, but more as a physicist, to point out a fundamental problem in the calculation of electric field strength distributions in the human brain. The modeling of the electric field strength distribution in this study was done according to a method that was used in a tDCS study [3]. This immediately points out an important problem, namely that the modeling was done with the basic assumption of a direct current application. This basic assumption is more than doubtful in several respects.

It is historically evident that ECT is an alternating current method, since sinusoidal wave generators were initially used. The sine waves were replaced by the modern short-pulse technology, but the frequencies used remained in the same spectrum. With an alternating current, however, it is not the electrical resistance that is crucial for Ohm’s law, as with a direct current, but the electrical impedance. However, the electrical impedance is not only determined by the electrical resistance, but also by inductivity and capacity of the current-carrying medium. These two parameters (capacity might be more important than inductivity), which are necessary for a correct modeling of the electric field, were not considered in the study. In addition, the portion of the electrical impedance determined by inductivity and capacity is strictly frequency dependent. In ECT, however, frequency typically differs depending on the applied charge (“dose”). This aspect is also not addressed in the study. Another very critical aspect is that the cited methodology study [3] uses current strengths of 1 mA for its calculations, as it is appropriate for tDCS. Currents used in ECT are 2–3 orders of magnitude higher, specifically 600–800 mA in this case. It is a well-known phenomenon in physics that much lower impedances are measured at higher currents (a so called “electrical breakdown” of impedance) [4]. Indeed this effect can also be observed with each individual ECT, because the so-called “static” impedance, which is measured before the stimulus current application, typically lies around 1000–2000 ohms, whereas during the current application the impedance is typically between 200 and 250 ohms. This effect, which would result in lower E-field strength (implicating that deeper brain structures would be less affected) is also not taken into account within the model. Strictly speaking, it must be noted that all these effects (inductivity and capacity, frequency dependence, and impedance dependence with current strength, and maybe even the waveform itself (e.g. sine wave vs. short rectangular pulses)) would have to be known as accurately as possible for each individual voxel of the brain to enable correct finite element modeling. Moreover, it is highly likely that strong direction-dependent effects, for example within the white matter tracts, play an additional role in the final modeling of the electric field [4, 5].

In summary, I would like to point out that the models for calculating the electric field distribution in the human brain should currently be interpreted with care, taking into account that they are not validated by direct measurements. In this regard, it would be desirable to first model electric field distributions in animals and then validate them by direct electric field measurements before implicating consequences for therapeutic decisions of ECT practitioners.
